# Dr. Corydon L. Ford

**Published:** 1895-08

**Authors:** 


"UNDER the lead of Dr. C. E. Stroud, of Sandusky, O., physicians over the country are being urged to assist in a movement to erect on the campus at the University of Michigan a bronze statue of the late Dr. Corydon L. Ford, America's greatest anatomist."

The proposition of the above paragraph is one eminently proper. Such a recognition of the service rendered by the late Prof. Ford, to the medical profession and to the Medical Department of the University of Michigan, and indeed to the entire University, and to several other medical colleges with which he as a teacher was associated, would seem to make it well-nigh imperative that some such recognition as indicated above should be made. Dr. Ford was for over forty years a teacher of anatomy, and for most of this time had not a superior, if an equal, in this country in this line of work. Thousand of physicians and dentists in this country, and indeed in other parts of the world, remember him with the warmest gratitude and heartiest respect, every one recognizing and acknowledging his superior ability. We here suggest that those who have been his pupils in the Dental Department of the University of Michigan bear this matter in mind and in their affections, and so soon as a call may be made for the means of carrying out the proposition, that every one be ready to respond in a manner worthy of the cause.

